# Role of primary surgery in advanced ovarian cancer

**DOI:** 10.1186/1477-7819-2-32

**Published:** 2004-10-02

**Authors:** Karsten Münstedt, Folker E Franke

**Affiliations:** 1 Department of Obstetrics and Gynecology, Justus-Liebig-University of Giessen, Klinikstrasse 32, D 35385 Giessen, Germany; 2 Institute of Pathology, Justus-Liebig-University Giessen, Langhansstrasse 10, D 35385 Giessen, Germany

## Abstract

**Background:**

Major issues in surgery for advanced ovarian cancer remain unresolved. Existing treatment guidelines are supported by a few published reports and fewer prospective randomized clinical trials.

**Methods:**

We reviewed published reports on primary surgical treatment, surgical expertise, inadequate primary surgery/quality assurance, neoadjuvant chemotherapy, interval debulking, and surgical prognostic factors in advanced ovarian cancer to help resolve outstanding issues.

**Results:**

The aim of primary surgery is a well-planned and complete intervention with optimal staging and surgery. Surgical debulking is worthwhile as there are further effective treatments available to control unresectable residual disease. Patients of gynecologic oncology specialist surgeons have better survival rates. This may reflect a working 'culture' rather than better technical skills. One major problem though, is that despite pleas to restrict surgery to experienced surgeons, specialist centers are often left to cope with the results of inadequate primary surgical resections. Patients with primary chemotherapy or those who have had suboptimal debulking may benefit from interval debulking. A proposal for a better classification of residual tumor is given.

**Conclusions:**

Optimal surgical interventions have definite role to play in advanced ovarian cancers. Improvements in surgical treatment in the general population will probably improve patients' survival when coupled with improvements in current chemotherapeutic approaches.

## Background

The Fédération Internationale de Gynécologie et d'Obstétrigue (FIGO) classifies ovarian carcinoma in stage I to IV [1,2]. Stage I has been defined as growth limited to the ovaries; stage II as growth involving one or both ovaries with pelvic extension; stage III as tumor involving one or both ovaries with peritoneal implants, and outside the pelvis and/or positive retroperitoneal or inguinal nodes and stage IV as having distant metastasis [1,2].

Tumors in stages I and II are generally considered to represent early disease, while stages III and IV evince late or advanced disease [3,4]. The strong prognostic value of the FIGO classification system has been proved in number of studies [5].

Unfortunately, most ovarian carcinomas are detected only when they are advanced. Results of studies evaluating screening by tumor markers (a raised CA125 value) and/or ultrasonography to detect early disease are not clear [6,7]. Ultrasonography may become an increasingly important tool as it has been associated with higher detection rates in early stage disease and in patients with a genetic predisposition to tumor [8-11]. However, use of proteomics will perhaps identify more ovarian carcinomas at early stages in the future [12].

Though controversial till 70s, surgery is now recognized an integral part of the treatment armamentarium in advanced ovarian carcinoma. Aure *et al*., [13] presented convincing evidence that extensive tumor removal resulted in better survival even in advanced stage disease and introduced the idea of primary tumor debulking surgery. The value of primary debulking surgery was confirmed and its theoretical background was elucidated by Griffith and Fuller [14]. Subsequent work showed that debulking surgery improves an adverse vegetative function and nutritional problems such as loss of appetite and nausea [15,16]. It was also suggested that primary debulking surgery removes therapy-resistant tumor cells and increases the number of proliferating tumor cells (the Gompertzian phenomenon), which makes these cells more susceptible to subsequent chemotherapy [17-19]. These early hypotheses have partly been confirmed by the finding of increased postoperative tumor proliferation rates in patients after surgery [20].

Ongoing discussions about quality assurance and guideline-based therapy had helped to foster the impression that the main issues in treating ovarian cancer have been resolved and that the value of each procedure involved has been supported by high levels of scientific evidence [21-23]. Closer inspection reveals that it is not true. Only a few treatment guidelines are supported by published reports, and even fewer by prospective randomized clinical trials. However, we strongly believe the value of retrospective studies is greatly underestimated. Recent analyses show that the treatment effects assessed by observational studies do not greatly differ in magnitude or quality from those published in randomized, controlled trials [24,25]. Furthermore, biases created by the selection criteria inherent in prospective randomized trials are frequently ignored.

Thus, in this article we will concentrate on looking more closely at several issues in surgical treatment, their effects and importance in relation to outcome in advance ovarian cancer.

### Primary surgical treatment

The utility of primary surgery for advanced ovarian cancer is well established. Its aim should be a well-planned, extensive and complete intervention. Thus, no facility should offer surgery for patients with ovarian cancer if adequate standards of care cannot be met.

The optimal preparation of patients for surgery is very important. Patients must be in a position to give fully informed consent to any additional surgical procedures found necessary during the operation. They should also undergo colonic lavage, which will provide the surgeon with better access to the lymph nodes and reduce risks in cases where intestinal surgery is undertaken. Where cytological evaluation of peritoneal fluid aspired preoperatively, a raised concentration of serum CA125, or ultrasound results indicate advanced malignancy, the patient should be transferred to a specialist surgeon (see below).

Many cases where the staging is not clear, laparoscopy appears to be good tool for obtaining a definitive histological diagnosis in advanced ovarian cancer and helps in planning the best surgical approach. Laparoscopic staging, in particular, can give a clear view of the extent of surgery required and the difficulties that may be expected, and it may be helpful in selecting patients for primary (neoadjuvant) chemotherapy [26]. Video recording can document the findings during laparoscopy and can be used subsequently by the surgeon to plan optimal debulking surgery.

However, laparoscopic surgery of any suspicious adnexal mass is not encouraged unless the risk of capsular rupture and tumor spill is minimized by the use of endobags [27]. No cystic mass which is >10 cm in diameter and/or adherent to the lateral pelvic wall should be removed laparoscopically [28].

The fear due to some *in-vitro *and animal studies that showed carbon dioxide pneumoperitoneum has adverse effects on outcomes is probably unfounded, as a recent analysis from second-look laparoscopies showed no influence of pneumoperitoneum on overall survival [29]. Certainly, more data on this issue is required.

Since laparoscopy may increase tumor growth rates, delays between laparoscopy and definitive surgery should be avoided [30]. Although this view has not yet been supported by any other study, we believe that the time between the suspected diagnosis of advanced ovarian cancer and surgery should be kept as short as possible. Delays may result in a higher preoperative tumor mass which has been identified as an adverse prognostic factor [31].

### Surgical staging

Tumor stage is one of the primary prognostic factors. Appropriate staging is vitally important for effective postoperative therapeutic decision-making. Patients who have been accurately staged as stage I may not require adjuvant chemotherapy [32,33].

Requirements for appropriate staging after total abdominal hysterectomy and bilateral salpingo-oophorectomy include multiple cytological washings, random biopsies from the peritoneum and the diaphragm, omentectomy and lymphadenectomy. The value of peritoneal cytology is supported by prospective studies [34]. There are several issues surrounding lymphadenectomy, and these are discussed later. In two studies, optimal staging resulted in 30% to 50% of the patients being reclassified to a higher stage – a fact which has implications for subsequent treatment [35,36]. A classification system for determining the quality of surgical staging was introduced recently and is shown in Table 1[33]. However, it may be only helpful for comparisons of older studies since optimal staging is a prerequisite of later therapeutic decisions.

**Table 1:** Surgical quality categories for staging of ovarian carcinomas (based on Trimbos et al. 2003)

**Category of surgical quality**	**Staging procedures included**
Optimal -	Inspection and palpation of all peritoneal surfaces; biopsies of any suspect lesion for metastasis; peritoneal washings; infra-colic omentectomy; blind biopsies of the right diaphragm and right and left para-colic gutter, pelvic side-walls of the ovarian fossa, of the bladder peritoneum and of the cul-de-sac and sampling of iliac and para-aortic lymph nodes
Modified -	Everything between optimal and minimal staging
Minimal -	Inspection and palpation of all peritoneal surfaces and the retroperitoneal area; biopsies of any suspect lesions for metastasis; peritoneal washing; infracolic omentectomy
Inadequate -	Less than minimal staging but at least careful inspection and palpation of all peritoneal surfaces and the retroperitoneal area; biopsies of any suspect lesion for metastasis

### Extent of surgery

In addition to the staging procedures mentioned earlier, optimal surgical treatment for ovarian cancer comprises tumor removal; removal of remaining ovaries, uterus, and fallopian tubes, omentectomy, and radical para aortic and pelvic lymphadenctomy [3,37]. The German national treatment guidelines recommend a simultaneous appendectomy and removal of the cul de sac over the peritoneum of the small pelvis [38]. Since removal of all grossly visible tumor is considered crucial for long-term survival, surgery should be extended to include hemicolectomy, splenectomy and stripping of the peritoneal reflection of the diaphragm when the tumor masses infiltrate the entire abdominal cavity, the colon, the diaphragm or other structures respectively.

Although the reasoning behind performing these measures seems convincing, only lymphadenectomy has been partly evaluated in a prospective, randomized trial. The previously held belief that mere palpation of lymph nodes is sufficient to gauge nodal status was refuted in this study [39].

Lymphadenectomy plays a triple role in the treatment of ovarian cancer. First, it is of diagnostic value since tumors of apparently early stage show nodal involvement in about 20% to 40% of the cases [40], If found positive, the tumor must be classified as stage IIIc. Secondly, lymphadenectomy is of immense prognostic value. Most importantly, lymphadenectomy may also have a therapeutic effect as retrospective studies comparing lymphadenectomy with no lymphadenectomy reported a survival benefits with this procedure [41-43]. The data on lymphadenectomy is however conflicting with one study showing that the patients with stage III disease (tumor residuals >2 cm) that has been debulked suboptimally do not benefit from lymphadenectomy [44]. Other workers report no benefit even if the residual tumor size is smaller (1 cm) [45]. Though not fully published, the only prospective, randomized trial shows that systematic lymphadenectomy did not result in better survival compared to selective lymphadenectomy [46]. Mainly based on the retrospective findings current views on treating stage III disease suggest: systematic lymphadenectomy in cases of residual tumors <1 cm, nodal debulking only where tumors are larger than intra-abdominal residuals, and nodal sampling in stage IV disease with pleural effusions only [47].

To the best of our knowledge, there has been no study testing the benefit of hysterectomy or omentectomy. However, the concurrent incidence of endometrial carcinoma in 10% to 25 % of patients, or its precursors in about 30% to 50% of all ovarian cancers justifies this procedure [48,49].

### Optimal debulking

Ovarian cancer is one of the tumors where surgical debulking is considered worthwhile. This is due to availability of further effective treatments that are available to control the unresectable residual disease. As early as 1934, Meigs suggested that maximum cytoreductive surgery was beneficial [50]. Many years later, in 1968, Munnell followed this idea and proposed the idea of 'maximum surgical effort' [51]. He distinguished between definitive surgery, partial removal of the tumor and biopsy only. Since partial removal covers a wide range of interventions that requires varying amount of efforts, optimal debulking was distinguished from suboptimal debulking. Although there is no generally accepted definition, most early studies considered a residual tumor size of <2 cm as optimal [52]. In a more recent survey among gynecological oncologists from United States of America (USA), 12% of the responders defined optimal debulking surgery as no visible tumor residuals, while 14% described it as residual tumor masses less than 0.5 cm. However, 61% chose a 1 cm threshold and 13% considered a tumor of 1.5 cm to 2.0 cm as optimal [53]. Comparative analysis of diameters of various residual diseases has shown that there exists some sort of a threshold at 2 cm, above which no significant differences in survival can be found. In contrast, subset analyses of smaller diameters in residual disease show improved patient prognosis [54]. This variation in the interpretation of thresholds with prognostic impact calls for a commonly accepted definition (see concluding remarks) and more controlled trials, that need to be non-randomized as it will not be ethically possible to leave some tumor behind.

The results of an earlier meta analysis on cytoreductive surgery might have been flawed not only due to absence of clear definitions but also due to the combined effects of subsequent chemotherapy [52]. In this study, the then novel, platinum-containing chemotherapy had a stronger impact on survival than cytoreductive surgery. A recent and otherwise comparable meta analysis however, confirms the greater survival benefit of patients undergoing maximum cytoreduction [55]. Another interesting study stated that optimal cytoreduction means no visible residual tumor [31]. It has been further shown that the volume of the residual tumor and the success of subsequent chemotherapy are interdependent [56].

### Expertise of gynecological oncology surgeons

Based on available evidence it is generally accepted that the experience and technical expertise of a surgeon are important prognostic factors. Comparisons of overall survival in patients treated by gynecological oncology, gynecologists and general surgeons have shown that patients treated by surgeons trained in gynecology (gynecological oncology) have a significantly better prognosis [57,58]. This finding may not reflect primarily on the technical skills of these surgeons but rather reflection the 'environment' in which they work – where views and thoughts on the biology of advanced tumors are freely shared and patients are often treated by a team rather then individuals. As for surgeons there are only select patients with uncommon neoplasms like gastrinomas, glucagonomas, stomatostatinomas, and VIPomas, who profit from debulking surgery with particular reference to prevent deleterious hormonal side-effects [59]. It is expected that new chemotherapy and immunotherapeutical approaches will probably lead to a re-evaluation of debulking surgery as a complementary approach [60-62]. Till such time where definite evidence is available, it is strongly recommended that all patients should be treated by a gynec-oncologist. There had been constant calls to regionalize specialist surgery, however, no studies have yet shown better survival in patients treated by 'high volume' operators or such specialists [63,64]. A recent study on quality control from Hesse, Germany, showed striking deficiencies even at central-referral hospitals [23]. Treatment by a multidisciplinary team of specialists, has been shown to increase patients' chances of survival without any disputes [65,66]. Patients probably benefit most from being treated in centers which promote excellent scientific exchange, and continuous education and self-evaluation among surgeons besides providing multidisciplinary approach to management.

### Coping with inadequate primary surgery

Surgical treatment of advanced ovarian cancer is one of the most demanding procedures in gynecological surgery. Despite repeated requests to restrict surgery to experienced surgeons, considerable numbers of patients are still operated by others. A population based study from Germany showed that omentectomies were performed in about 50% of all cases of ovarian cancer and lymphadenectomy were carried out only in 30% [22]. Another study from USA showed that only about half of the patients receive 'standard' care [68]. In spite of the establishment of gynecological oncology as a specialty in the USA, fewer than half of the patients were originally seen by such a specialist [68]. More over the terminologies like "standard" are not defined well.

The situation with regard to specialism depends strongly on the medical infrastructure, and varies from country to country and region to region. However, the problem of inadequate primary surgery is real, and coping with it is a frequent task in specialist centers even in developed countries. The question is what should be done for the patient concerned? Interestingly, this is something that cannot be found in textbooks [3,69,70]. Some of the literature suggest re-laparotomy by experienced surgeons to achieve reductions in all possible tumor mass [71,72] however, there is no evidence to support this strategy.

In general, one of two situations occurs. First patients present with no evidence of macroscopic tumor residuals but staging procedures and/or operative measures were omitted. Computed tomography (CT) or Magnetic resonance imaging (MRI) to identify enlarged lymph nodes or possible residual tumors in these situations may help to decide on the need for second surgery. The belief that tumor cells in retroperitoneal lymph nodes are better able to survive chemotherapy is supported by low cytotoxic drug concentrations in these [73]. Therefore, it is reasonable to consider enlarged lymph nodes as a decisive factor favoring a direct surgical approach. It is interesting to note that in endometrial carcinomas, a clinically negative omentum was also found to be histologically negative in most cases (sensitivity 89%) [74].

Patients with residual tumor mass have to be evaluated to determine if it is possible to achieve no residual tumor or microscopic residual tumor by immediate secondary surgery. Although immediate laparotomy seems to be a good idea, the limited capacity for surgery at specialist departments and delays in having the surgery are to be considered. The peritoneum shows inflammation shortly after surgery reaching a high about 7 to 14 days afterwards [75]. Surgery at this time is considered far more complicated and may result in higher blood loss and greater risk of injury to neighboring abdominal organs [76]. However, waiting for the inflammatory processes to resolve will give the tumor further time to proliferate [20]. Therefore, interval debulking surgery after three courses of chemotherapy should be considered as an appropriate alternative.

### Neoadjuvant chemotherapy

Disease spread >2 cm to the spleen, diaphragm, liver surface, mesentery, or gallbladder is generally believed to be inoperable. However, even these patients may often undergo effective debulking procedures [26,77]. As mentioned earlier, laparoscopy can be used to reach decisions on surgery. The only problem with laparoscopy in combination with neoadjuvant chemotherapy is a 30% rate of port site metastasis, which is believed to be a result of the pneumoperitoneum procedure created for laparoscopy [78]. These metastases should be excised at the time of any subsequent surgery [79].

An analysis of several retrospective studies on neoadjuvant chemotherapy showed that there are no good reasons to assume that this approach is associated with a poorer prognosis [26]. The European Organisation for Research and Treatment of Cancer (EORTC) protocol 55971 comparing upfront tumor debulking surgery with neoadjuvant chemotherapy in patients with stage IIIc or IV disease is accruing and its results will provide better insights on this issue.

### Interval tumor debulking

Interval debulking, is another approach to reduce tumor burden between the cycles of chemotherapy. It has been evaluated in two prospective randomized trials [80,81]. The EORTC study showed a clear survival advantage for interval debulking (still noted in the 2001 update), the Gynecologic Oncology Group (GOG-152) study has, as yet, failed to show any benefit from interval debulking [80,81]. Patients with residual tumor >1 cm received three courses of cyclophosphamide/cisplatinum in the EORTC study [80] or three courses of paclitaxel/cisplatinum in the GOG-152 study. In both studies, patients who did not respond to chemotherapy were removed from the study. Those who responded were randomized to either secondary surgery or no surgery. Afterwards, all patients received three more courses of the earlier chemotherapeutic regimen.

Although there seem to be only minor differences in the design of both trials, a closer look shows that in the GOG-152 study, the number of stage IV patients was lower (6%) compared with the EORTC (21%) study, the performance status was better, and there was less residual tumor. This was due to the eligibility criteria for GOG 152 which stated that patients should have had surgery with maximal effort to resect the uterus, tubes, ovaries, omentum, and all gross residual ovarian cancer at the time of primary surgery.

The questions about the benefit of interval debulking surgery remain unresolved. However, it appears that patients who have had neoadjuvant chemotherapy or suboptimal debulking may profit from this treatment, while those who have undergone primary, maximum effort surgery by a gynecological oncologist are less likely to profit from it [82].

### Surgical prognostic factors

Size of the residual tumor, volume of the residual disease and experience of the surgeon are important prognostic factors [56,77]. Among these, only few can be influenced by human intervention. Except for dose intensity of chemotherapy, recent literature indicates that the hemoglobin concentrations before chemotherapy are of prognostic value [83,84]. The latter may be influenced by the use of erythropoietin which has shown positive effects on survival in cervical cancer [86].

As mentioned above, the frequently used definition of <2 cm for optimal debulking is arbitrary since every further reduction in the size of residual tumor improves the prognosis [54]. Thus, each threshold between 0 and 2 cm will have its own prognostic relevance. While the criterion 'diameter of residual tumor' reflects tumor cell hypoxia and reduces the pool of proliferating tumor cells susceptible to chemotherapy, the criterion 'residual tumor volume' alludes to the removal of therapy-resistant tumor cells, which are believed to be responsible for early recurrences. A comparison of both criteria in relation to their prognostic impact has shown that residual tumor volume is of greater importance [56]. As shown in subgroup analyses of the intergroup trial confirming the results of the GOG-111 study, a possibly superior chemotherapeutic regimen containing taxanes cannot compensate for the tumor left behind after primary surgery [19].

### Future trends

It is difficult to assess the future role of surgery in advanced ovarian cancer. Neoadjuvant chemotherapy may become more important. However, as the tumor debulking surgery works, except in stage IV patients with solid distant metastasis, it may be worth trying combined ultrasound-guided laser interstitial thermotherapy for non-resectable liver metastasis with conventional debulking surgery [87]. Apart from technical innovations, quality control, quality assurance and documentation of patient outcomes after surgery will probably play major parts in treatment improvements.

## Conclusion

Numerous studies have analyzed the effects of various kind of chemotherapy in ovarian cancer. In contrast, only a few prospective randomized studies have focused on surgical issues in this type of tumor [46,80,81]. This lack of surgical trials has probably contributed to inhomogenous definitions regarding the terminology of surgical interventions and surgical stages (early compared with late) and the classification of operative success in general.

In future, we must aim to ensure that all patients are treated along the generally accepted guidelines and receive optimal debulking surgery which leaves only microscopically detectable residual tumor as shown in number of studies; it is certainly unethical at present to evaluate this procedure in randomized clinical trials (see Introduction). Other then this, there are many other relevant issues which need to be resolved or clarified with special reference to neoadjuvant chemotherapy, interval debulking, the surgeon's training, and inadequate primary treatment. It would certainly be very helpful to demonstrate clearly the consequences of what is supposed to be an inadequate treatment.

The simple dichotomization of FIGO stages to early or late does not correspond to any diagnostic, biological or therapeutic advantage. We believe that it is far more reasonable to consider stage I alone as early disease (perhaps even only stage Ia and b), stages II and III (perhaps even stage IV with pleural effusions) as intermediate disease, and stage IV with organ metastasis as advanced disease. Such a classification would follow current views on treatment, since early ovarian carcinomas are treated primarily by surgery (eventually fertility-sparing) and adjuvant chemotherapy in cases of increased risk, intermediate ones by surgery and routine chemotherapy, and advanced disease by chemotherapy only. Thus far, results of studies on stage IV patients with organ metastases are inconsistent regarding the benefit of surgery. This is another issue to be resolved [88]. In this respect, FIGO may find subdividing stage IV into stage IVa (pleural effusions) and IVb (organ metastasis) worthwhile.

Disappointingly, the Tumor-Nodes-Metastasis (TNM) classification follows the FIGO system and violates its own principles by not accepting distant peritoneal metastasis as a natural indicator of primary tumor size but also by summarizing a nodal-positive disease stage as T3c (corresponding to FIGO IIIC) regardless of intra-abdominal findings. This inconsistency has already created curious confusions in current research. Nodal involvement has been shown to impair prognosis, however, smaller intra-abdominal tumors with nodal involvement (presented as stage I to IIIb disease but, by definition, all stage IIIC) show a significantly better prognosis than extensive intra-abdominal tumor masses (again stage IIIC) [89].

Furthermore, residual disease should be properly defined. The most rational approach is to regard microscopic residuals as optimal. Case series claim that experienced gynecologic-oncologic surgeons can clear up to 85% of patients in the unfavorable subgroups (FIGO stage IIIc and IV) of all visible tumor, leading to an extraordinarily high five year survival rate of about 50% [77]. As discussed (see surgical prognostic factors), a good definition of residual tumor would include aspects of both residual tumor size and volume. A proposal is made in Table 2.

**Table 2:** Surgical documentation of residual tumor after debulking of ovarian carcinomas

Residual tumor status*	**Maximum diameter of residual tumor**	**Maximum total volume of residual tumor**
Optimal	Microscopic	No visible tumor
Minimal	< 1 cm	≤ 10 cm^3^
Intermediate	1 – 2 cm	> 10 cm^3 ^but ≤ 100 cm^3^
Gross	> 2 cm	> 100 cm^3^

*To assign residual tumor to a certain status, both criteria, diameter and volume, have to be fulfilled. Otherwise the next lower category should be used.

In summary, more attention need be paid to surgery for advanced ovarian cancer. These include necessary improvements in treatment in the general population, uniform definitions and terminology, and increasing number of surgical clinical trials. Extrapolating from the results of truly optimal ovarian cancer surgery, we believe that improvements in surgery will lead to better patient survival than improvements in current chemotherapeutic approaches [55,90,91].

## Competing interests

The authors declare that they have no competing interests.

## Authors' contributions

KM and FEF both participated equally in literature search, conceptualization and preparation of the manuscript. Both authors have read the manuscript and approve it for publication.
